# An ERP Study of Modality-Specific Effects on Emotional Word Processing

**DOI:** 10.3390/brainsci16050488

**Published:** 2026-04-30

**Authors:** Yue Huang, Xiaogen Liao, Chuanbin Ni

**Affiliations:** 1 School of Foreign Languages and Cultures, Nanjing Normal University, Nanjing 210024, China; huangyue1110@163.com; 2 College of Liberal Arts, Nanjing University of Information Science and Technology, Nanjing 210044, China; junteng0510@163.com

**Keywords:** modality-specific effect, emotional valence, emotional words, interoception

## Abstract

**Background/Objectives:** Sensory experiences and emotional information contribute to conceptual knowledge. Compared to exteroceptive modality (e.g., visual, auditory), interoceptive modality predominates in the representation of emotional concepts. However, few studies have examined the interoceptive modality-specific effects on emotional word processing. Additionally, questions remain about when emotional valence interacts with sensory experiences during the processing of emotional words, and to what extent these words are grounded in different sensory experiences. **Methods:** To address these gaps, the present ERP study investigated how sensory information (interoception and vision) influences emotional word processing in a lexical decision task. **Results:** Behavioral results showed significant differences between interoceptive and visual words, as well as between positive and negative valence. A trend toward an interaction between sensory modality and emotional valence was also observed. ERP results indicated that negative words elicited a more positive-going P2 than positive words. Significantly smaller N400 amplitudes were observed for interoceptive words than visual words in the positive condition. Negative visual words evoked enhanced LPC amplitudes compared with both negative interoceptive words and positive visual words. **Conclusions:** The present findings suggest a dynamic pattern of valence effects in emotional word processing, characterized by a negativity bias and a positivity bias at different stages. Furthermore, our findings highlight that interoception promotes the semantic retrieval and integration of emotional words. This study provides empirical support for the modality-specific hypothesis within the framework of interoceptive embodied cognition and offers novel implications for future research on emotional word processing.

## 1. Introduction

According to embodied cognitive theory, embodied experience shapes language and cognition [1]. Language plays an important role in how people perceive and express emotions. Emotional words not only can evoke corresponding emotional states, but also activate embodied experiences. They are grounded in the sensorimotor system and represented through perceptual simulations [2]. Thus, sensorimotor experience and emotional information jointly influence lexical processing [3]. However, it remains unclear how and to what extent sensorimotor experiences modulate the representation of emotional concepts.

Recently, interoception—defined as the perception of physiological states within the body [4]—has been considered as a turn of embodied cognition. From an interoceptive perspective of embodied cognition, physiological signals are processed by the interoceptive system before being relayed to the brain, positioning interoception as a critical bridge between the body and the mind [5]. Interoception centers on internal bodily states, distinguishing it from exteroception, which refers to the sensations from outside the body [6]. Recent advances in interoceptive embodied cognition provide evidence for embodied representation of emotional concepts. For instance, Jiang et al. [7] reported that processing Chinese affective verbs related to interoceptive reaction and exteroceptive reaction was modulated by aging. Behavioral results indicated that older adults responded more slowly to interoceptive verbs than to exteroceptive verbs, whereas positive words were processed faster than negative words in both interoceptive and exteroceptive conditions. Additionally, a growing body of studies using sensorimotor norms rating paradigms has further demonstrated that interoceptive experience is as important as exteroceptive experience for semantic knowledge and plays a pivotal role in emotional concept representation [8,9,10,11]. For example, Zhong et al. [11] identified that Chinese emotional words with interoceptive perceptual strength contain morphemes or radicals referring to visceral organs, and most such words encode emotion-related metaphors via visceral sensations. Nevertheless, the relationship between interoception and emotional valence receives less attention. Moreover, although sensorimotor norm studies have reported a negative correlation between interoceptive and visual modalities, few investigations have directly compared processing differences between interoceptive and visual information during emotional word recognition.

Prior work argued that conceptual knowledge is grounded in modality-specific perceptual experiences. Using functional magnetic resonance imaging (fMRI), González and colleagues [12] found that reading scent-related words (e.g., garlic, cinnamon) activated the primary olfactory regions in the brain, indicating that conceptual knowledge of such terms is grounded in olfactory experience. Collectively, these findings suggest that conceptual representation relies on the simulation of perceptual experiences and the reactivation of brain regions involved in corresponding sensory processing [8]. However, many studies have focused on visual modality or comparisons between motor and visual modalities [13,14], or between visual and tactile modalities [15]. Harpaintner et al. [13] first reported that the brain activation to abstract concepts related to motor (e.g., fitness) is different from visual feature content (e.g., beauty) in the lexical decision task. fMRI results showed the increased activation of the frontal and parietal motor areas for motor abstract words, and the temporo-occipital visual areas for visual abstract words. This suggests that abstract concepts are grounded in modality-specific brain systems. The conceptual feature content determines the engagement of perceptual and action systems, extending the grounded framework of conceptual representation to sensorimotor brain networks. Subsequently, Harpaintner et al. [14] used event-related potentials (ERPs) to examine the temporal dynamics of similar stimuli and found significant differences between motor and visual words at the early period, reflecting rapid access of modal features during conceptual processing. Connell and Lynott [15] observed a tactile processing disadvantage during conceptual processing. Together, these studies support the view that the conceptual system recruits modality-specific perceptual systems for representation.

In the same vein, several studies have investigated emotional features across sensory modalities in word processing (e.g., [16,17,18]), with the notable exception of interoception. For instance, Winter [18] found that words related to taste and smell modalities (e.g., pungent, sweet) had stronger emotional valence compared to other senses, based on large-scale sensorimotor and valence norm datasets. At the neural level, Herbert [16] observed early ERP modulations by the emotional valence that were independent of word class or task. These findings support that the sense-related words are associated with specific emotional features and that sensory information and valence jointly have impacts on the processing speed of words. Theoretically, the modality-general hypothesis posits that, regardless of the modalities that elicit the emotions, valence states are consistently represented. By contrast, the modality-specific hypothesis claims that valence states are represented specifically depending on the modalities [19]. To answer this debate, prior work investigated the patterns of valence processing elicited by different modalities (e.g., visual and auditory). Gao et al. [20] revealed modality-specific valence effects, showing that valence influences word recognition differently in visual versus auditory modalities. Analyses of lexical decision times across multiple large databases revealed that positive valence facilitated recognition in the visual modality, whereas emotional content generally facilitated processing in the auditory modality relative to neutral content. These findings indicate that valence effects on lexical processing are modality-dependent. Despite these advances, research on the modality specificity of interoception during emotional word processing remains scarce and inconclusive. Accordingly, the present study aimed to further clarify this issue.

The foundational role of interoception in emotional experience can be traced back to the embodied emotion views. The Peripheral Theory of Emotion posits that emotions originate from physiological changes [21]. The somatic markers hypothesis further proposes that the core of the subjective experience of emotions consists of perceiving the pattern of changes in the body, and representations of emotions include the somatosensory component [22]. Correspondingly, Daikoku et al. [23] argued that emotional feelings and their bodily representations are not simply determined by physiological inputs, but emerge from the dynamic interaction among bodily signals, behavioral processes, and conceptual systems. Bodily signals include bottom-up physiological inputs such as interoception and autonomic responses. Given that interoception contributes to emotional experience, the modality specificity of interoception in emotional word processing warrants investigation.

No consensus has been reached concerning which valence has processing advantages. A substantial body of empirical work on emotional effects agreed that emotional stimuli have processing advantages over neutral stimuli, as emotional information influences semantic processing [24,25]. Many ERP studies revealed that emotional features modulate ERP components at distinct processing stages (e.g., [26,27,28,29]). Several EPR components are known to reflect emotional word processing according to the relevant literature. In the early stage, the early posterior negativity (EPN) is typically observed around 200 to 300 ms after stimulus onset with a temporo-occipital distribution, and its amplitude is more sensitive to emotional features than neutral ones, reflecting enhanced attention toward emotional stimuli [30]. The EPN effect is commonly modulated by emotionality [31,32]. This component is thought to reflect automatic processing of emotional stimuli or selective attention [33]. Another similar component, P2 over the fronto-central sites, indexes automatic attention capture during the early stages of meaning encoding, and its time window is similar to that of the EPN component. Previous studies also observed emotional effects in the P2 component. For instance, Herbert et al. [34] found that pleasant and unpleasant adjectives evoked larger amplitudes of P2 than neutral ones, whereas Zhang et al. [29] found that negative stimuli elicited a larger P2 component than positive and neutral ones. By contrast, Zhao and Guo [35] reported more positive-going P2 effects for positive words. During middle and later processing stages, the N400 and LPC components have been associated with the semantic integration and sustained analysis of emotional words in prior research [30]. Specifically, the N400 component peaks between 300 and 500 ms with a typical distribution among frontal and centro-parietal sites, reflecting the efforts for semantic retrieval and integration. The existing evidence suggests a reduced N400 for emotional stimuli compared to neutral words [26,32,35,36]. The LPC component, generally peaking between 500 and 800 ms over centro-parietal sites, displays sustained elaborate evaluation of emotional contents. Importantly, prior work found that the LPC amplitudes are different in response to positive words from negative words [29,37], indicating that it can discriminate emotional valence. Given that emotional valence is such a crucial factor for the representation and categorization of human experience, it is worth exploring the differences between positive and negative stimuli. Although there has been a consensus on the emotional effects in word processing, fewer efforts are devoted to examining the asymmetries in the way individuals use positive and negative information [38,39] or the discrimination between positive and negative words [40]. Since sensorimotor experience and emotional information contribute to word processing, whether sensory information modulates the valence processing, leading to a positivity bias or a negativity bias, awaits exploration.

In summary, interoception fundamentally shapes emotional experience [23], which provides a sensory basis for emotional concepts. Given that emotional concept representation relies on this internal sensory modality [41], interoception is considered a predictor of the lexical processing performance [8,11]. Nevertheless, how interoception influences emotional word processing warrants exploration.

To fill these gaps, the present ERP study investigated the effects of sensory information (i.e., interoception and vision) on emotional word processing in a lexical decision task. Furthermore, we aimed to examine whether modality-specific effects modulate emotional valence from an interoceptive embodied cognition framework. Existing research highlights the role of sensorimotor experience in semantic processing and suggests that interoception dominates emotional concept representation. We therefore hypothesized that interoceptive and visual modalities would differ in emotional word processing. Although few prior studies have examined interactions between interoception and valence during lexical processing, we predicted that embodied experience would interact with emotional valence and that interoceptive information would modulate valence effects during emotional word processing. To our knowledge, this is the first ERP study to directly compare interoceptive and visual modalities during emotional word processing and to clarify how interoception modulates emotional valence.

## 2. Methods

### 2.1. Participants

Forty Chinese Mandarin speakers (9 males and 31 females, mean age = 23.4, age range = 19–27) were recruited for the experiment. A minimum sample size of 24 participants, for our current research design, was calculated by G*Power 3.1 (Effect Size = 0.25, *α* = 0.05, Power = 0.8) [42]. All participants had an average of 16.45 years of education (SD = 2.05). They declared no history of neurological or psychiatric disorders and reported being right-handed, and having normal or corrected-to-normal vision. During offline data analysis, seven participants (1 male and 6 females) were discarded due to excessive number of artifacts in their electroencephalogram (EEG). After receiving the relevant information about this experiment, they signed the written informed consent, which was approved by the ethics committee of the university. They were reimbursed for their participation.

### 2.2. Design and Materials

The experiment employed a 2 (sensory information: interoception, vision) × 2 (emotional valence: positive, negative) within-subject design.

A total of 120 emotional words were determined for the current experiment and divided into four conditions (30 words each): positive interoceptive words, positive visual words, negative interoceptive words, and negative visual words. All Chinese stimuli were the translation equivalents of the English emotional words selected from an established database [10], which includes sensorimotor ratings for 40,000 English words across six sensory modalities (e.g., vision, hearing, smell, taste, touch, and interoception). Firstly, emotional adjectives dominated by either interoceptive or visual modalities were selected. Next, three Chinese postgraduates were recruited to translate English materials into Chinese two-character words, and another three postgraduates used back-translation, following Chen et al. [36]. Then, twenty college students who did not take part in the experiment rated the familiarity of each word on a 7-point Likert scale (1 = very unfamiliar; 7 = highly familiar). For familiarity and strokes, there were no significant differences across the four conditions (*ps* > 0.05). Frequency and concreteness were matched across conditions using the SUBTLEXCH corpus [43] and concreteness database [44] (*ps* > 0.05). Additionally, emotional valence and arousal of each word were confirmed through a database [45]. Arousal was not significantly different across the conditions (*ps* > 0.05), but positive words had higher valence than their negative counterparts (*p* < 0.001).

Another 120 Chinese pseudowords were generated as fillers. Each pseudoword consisted of two characters that followed orthographical and phonological standards (e.g., 扰淮 and 尖宙). We created these pseudowords following Chen et al. [36] and Ye and Zhao [46]. Strokes were matched between the real word and pseudowords (*p* > 0.1). Descriptive statistics of stimuli are presented in Table 1.

### 2.3. Procedure

**Figure 1:**
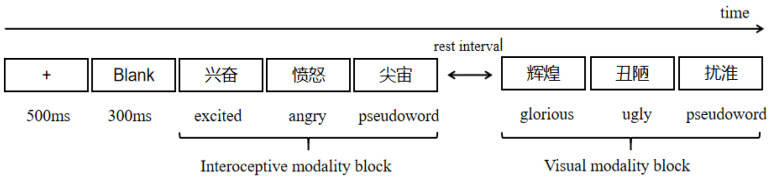
Experimental procedure.

The procedure was programmed by E-prime 2.0 software. The formal experiment consisted of 6 blocks. Each block included 20 emotional words (10 positive words, 10 negative words) from the same sensory experience and 20 pseudowords to minimize the cognitive load and potential modality-switching cost. The blocks were counterbalanced among participants and presented pseudorandomly such that consecutive blocks contained different sensory modalities. The trials were randomly presented within each block. Each trial began with a 500 ms fixation, following a 300 ms blank. Then the target word lasted for 1500 ms. Participants were required to judge whether the stimuli were real words or non-words shown on the screen by pressing the keyboard as quickly and accurately as possible. If it was a real word, pressed “A” with left index finger, if non-word, pressed “L” with right index finger, then followed by a 500 ms blank. Prior to the formal experiment, participants were seated in a relaxing state and got ready for a practice with 16 trials in order to familiarize themselves with the procedure. The whole experiment took nearly 60 min including electrode preparation. All participant were reimbursed for their participation. The experimental procedure is seen in Figure 1.

### 2.4. EEG Recording

**Figure 2:**
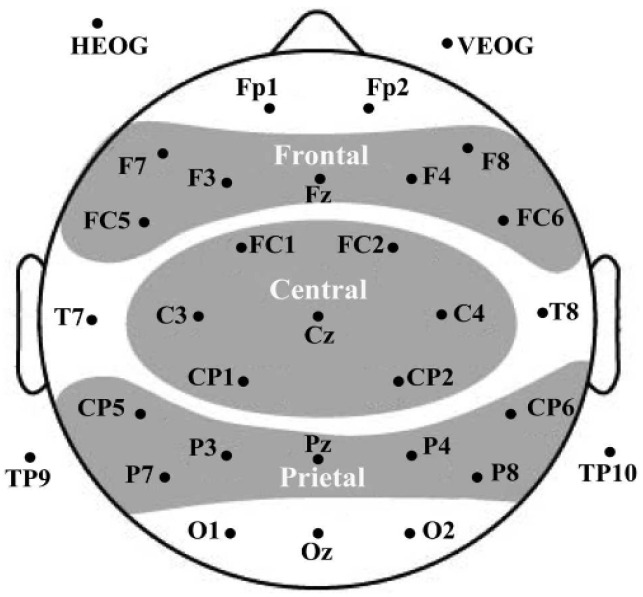
Electrode configuration.

**Figure 3:**
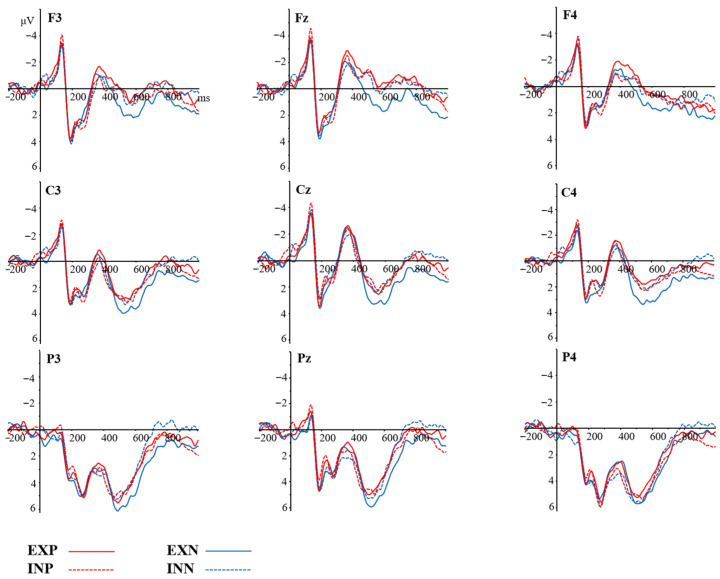
Grand average ERP response to four types of words. Note: EXP = exteroceptive positive; INP = interoceptive positive; EXN = exteroceptive negative; and INN = interoceptive negative.

**Figure 4:**
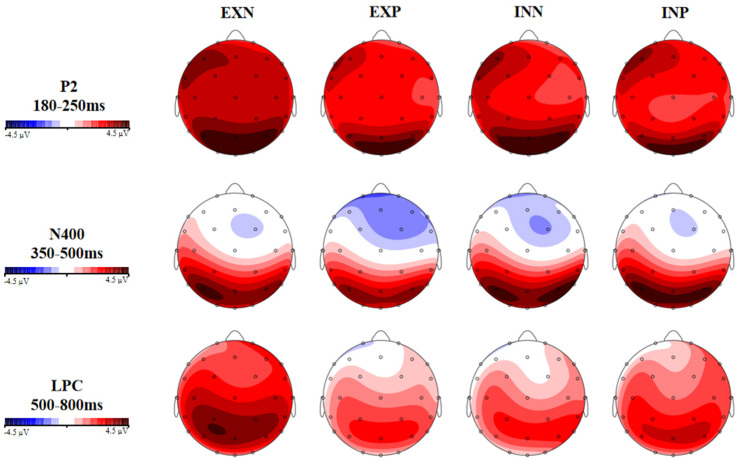
Topographic maps for the P2, N400, and LPC components across four types of words. Note: EXP = exteroceptive positive; INP = interoceptive positive; EXN = exteroceptive negative; and INN = interoceptive negative.

EEG data were recorded using a 32-channel active electrode cap (Electro-Cap International, Inc., Eaton, OH, USA) at a sample rate of 500 Hz. Electrode impedance was maintained below 5 kΩ. The vertical and the horizontal electrooculogram were recorded via two electrodes, which were placed above the right eye and at the outer canthus of the left eye, respectively. Offline EEG data were analyzed using Brain Vision Analyzer (version 2.1; Brain Products, Munich, Germany). Raw data were re-referenced to the left and right mastoids and were filtered offline between 0.1 and 30 Hz (24 dB/oct) using a basic FIR filter. Independent Component Analysis (ICA) was adopted to remove ocular artifacts. Artifacts exceeding ±100 μV (bad epochs) were automatically discarded, resulting in a loss of 15.36% of trials per condition, on average. The valid trials were no fewer than 20 per condition in every participant. Epochs ranging from −200 ms to 1000 ms after the onset of the stimulus were averaged for the four word types. All epochs were corrected to a 200 ms pre-stimulus baseline. Figure 2 is the electrode configuration. Figure 3 shows the ERP waveforms of mean amplitudes at selected electrodes elicited by four types of words. Figure 4 presents topographic maps across conditions.

Based on the previous studies [14,27,34,47] and after visual inspection of the wave-forms and topographic maps in this study, three ERP components were selected to investigate the effects of sensory information and valence in abstract word processing. As for P2, the mean amplitude of six electrodes was computed (CZ, C3, C4, PZ, CP1, CP2) within the time window of 180–250 ms. Mean amplitude of N400 was scored at four electrodes (FZ, F3, F4, FC2) within the time window of 350–500 ms, and LPC mean amplitude was scored at seven electrodes (CZ, C3, C4, CP1, CP2, PZ, P3) within the time window of 500–800 ms.

### 2.5. Behavioral and ERP Data Analysis

Reaction times (RTs) were analyzed using linear mixed-effects modeling with the lme4 package [48] in R [49]. Two fixed effects were considered: sensory information (interoception, vision) and valence (negative, positive). The model was fit using the maximal random effects structure that converged [50]. If the model failed to converge, we removed small variance parameters until the model adequately fit the data. The incorrect responses (1.13%) and outlier trials (±2.5 SD) (2.82%) for each participant were not analyzed.

All ERP data were analyzed using IBM SPSS Statistics v22. Two-way repeated measures ANOVA on the mean amplitudes of P2, N400 and LPC components was conducted with sensory information and valence as within-subjects variables. The Greenhouse–Geisser correction was adopted for *p*-values. Bonferroni correction for multiple comparisons was applied in post hoc pairwise comparisons.

## 3. Results

### 3.1. Behavioral Results

The mean accuracy for four types of words exceeded 98.8%, indicating that participants understood the task and performed well (M_EXN_ = 98%, SD = 0.02; M_EXP_ = 98.7%, SD = 0.02; M_INN_ = 99.1%, SD = 0.02; M_INP_ = 99.5%, SD = 0.01).

Linear mixed-effects regression revealed significant main effects of sensory information (*β* = −0.037, *SE* = 0.017, *t* = −2.267, *p* = 0.025) and valence (*β* = −0.037, *SE* = 0.017, *t* = −2.236, *p* = 0.027). This indicated that reaction times were shorter for interoceptive words (M = 606, SD = 133) than for visual words (M = 615, SD = 127), and faster for positive words (M = 606, SD = 128) than for negative words (M = 616, SD = 131). The sensory information-by-valence interaction was marginally significant (*β* = 0.042, *SE* = 0.023, *t* = 1.795, *p* = 0.075), showing a trend toward processing advantages of interoceptive words compared to visual words in the negative condition (*β* = 0.037, *SE* = 0.017, *z* = 2.267, *p* = 0.023). Additionally, the valence effect was significant in the vision condition, with positive words processed faster than negative words (*β* = 0.037, *SE* = 0.017, *z* = 2.236, *p* = 0.025). No other significant effects were found (*ps* > 0.05).

### 3.2. ERP Results

#### 3.2.1. P2 (180–250 ms)

The main effect of valence was marginally significant (*F* (1, 32) = 3.969, *p* = 0.055, *η*^2^ = 0.11), showing that negative words (3.083 μV) evoked greater positivity than positive words (2.562 μV) at central and parietal sites. No other significant effects were found (*ps* > 0.05).

#### 3.2.2. N400 (350–500 ms)

The main effects of sensory information (*p* = 0.53) or emotional valence (*p* = 0.572) were not significant. A trend toward an interaction between sensory information and emotional valence was observed (*F* (1, 32) = 3.174, *p* = 0.084, *η*^2^ = 0.09). Post hoc comparisons indicated that interoceptive words (−0.22 μV) elicited significantly smaller N400 amplitudes than visual words (−1.218 μV) in the positive condition (*p* = 0.047). No other effects were found (*ps* > 0.05).

#### 3.2.3. LPC (500–800 ms)

The main effects of sensory information (*p* = 0.272) or emotional valence (*p* = 0.205) were not significant. There was a significant interaction between sensory information and emotional valence (*F* (1, 32) = 5.194, *p* = 0.029, *η*^2^ = 0.14). Post hoc comparisons indicated that visual words (3.274 μV) elicited a larger LPC than interoceptive words (1.875 μV) in the negative condition (*p* = 0.064). Moreover, in the vision condition, negative words (3.274 μV) elicited enhanced LPC amplitudes compared to positive words (1.878 μV) (*p* = 0.024). No other effects were observed (*ps* > 0.05).

## 4. Discussion

The present ERP study investigated the modality-specific effects on the processing of emotional words. Behavioral results revealed significant disparities between interoceptive modality and visual modality and between positive words and negative words. Moreover, there was a trend toward an interaction between sensory information and emotional valence. ERPs results demonstrated that negative words elicited more positive-going P2 than did positive words over central-parietal sites. A smaller N400 was elicited by interoceptive words than visual words in the positive condition, over the frontal area of the brain. A greater LPC was evoked by negative visual words than negative interoceptive words and positive visual words over the central-parietal area. Combined with existing results in this study, the modality-specific effects on emotional word processing were discussed from an interoceptive perspective within the framework of embodied cognition.

### 4.1. Dynamic Pattern of Valence Effects in Emotional Word Processing

Behavioral and ERP results demonstrated a positivity bias and a negativity bias in emotional word processing, respectively. At the behavioral level, positive words were responded to quickly relative to negative words, which is consistent with previous studies (e.g., [39,51]). Our findings align with the broaden-and-build theory of positive emotions, which posits that positive emotional experiences temporarily broaden attention, cognition and behavioral repertoires in the moment, and gradually facilitate the accumulation of durable, transferable personal resources across physical, intellectual, social and psychological domains over time, ultimately helping individuals make optimal decisions [52]. Words containing positive information are more easily encoded and stored in memory; when retrieved again, they are activated and understood faster [41]. Another possible explanation comes from Kauschke et al. [39], who proposed a “negative delay” to describe the delayed disengagement of attention and longer reaction times for processing negative stimuli compared to positive stimuli. Accordingly, the observed behavioral positive advantage may also arise from sustained disengagement from negative stimuli. Remarkably, positive visual words were processed faster than negative visual words, indicating that a positivity bias was pronounced in visual modality compared to interoceptive modality. This suggests valence effects are modulated by sensory information in word processing. Consistently, prior work argued that the emotional modulation of word recognition is modality-specific [20], which found that the more positive the valence of the word, the faster the lexical decision time, reflecting a positivity bias in both visual and auditory modalities. Notice that our study extends the findings of Gao et al. [20]. Firstly, the present study examined the relationship between modality-specific sensory experiences (shaping semantic knowledge of emotional concepts) and valence, whereas Gao et al. [20] focused on valence effects in the processing of emotional words presented in different sensory modalities (i.e., written or spoken words); secondly, our study provides ERP evidence for the modality specificity of valence effects, complementing prior database-driven behavioral findings.

Furthermore, our findings revealed a negativity bias in the neural processing of emotional words. More positive amplitudes of P2 were elicited by negative words than positive words, regardless of sensory information. P2 indexes an early automatic attention capture by emotional information [30]. As suggested by Zhang et al. [37], the brain allocates early attention to differentiate the non-threatening from potentially threatening information. In line with Gao et al. [53], negative stimuli demand more attention and cognitive resources as an evolutionary adaptive mechanism for humans to avoid potential risks [54]. In addition, the LPC effect was modulated by an interaction of valence-sensory information, with negative visual words eliciting larger LPC amplitudes than positive visual words. This indicated that negative stimuli associated with vision underwent more sustained and elaborate processing at the last post-lexical stage. These findings were consistent with prior work [31,35], reporting that the LPC responses to negative words were augmented more than positive ones, although Zhang et al. [37] found a positivity bias in the LPC component, reflecting a “positivity offset”. It is worth noting that a negativity bias emerging in both LPC and P2 components reflects that, at the neural level, negative content not only captures attention at the early stage but also recruits additional cognitive resources for elaborated processing. During post-lexical processing, recognizing emotional words activates corresponding sensory experiences. For visual words, it involves simulating and constructing complex visual situational information during mental representation, which increases LPC amplitudes. A plausible account, proposed by Harpaintner et al.’s study [14], is that reading visual words increases the demand for the retrieval of more imaginable conceptual information. Nonetheless, in addition to the sensory basis of words, our study incorporated emotional valence as a key modulating factor, which extends the prior work [14].

Collectively, this study reveals a dynamic pattern of valence effects during emotional word processing, as indexed by existing behavioral and ERP findings. Firstly, negative stimuli capture the early attention compared to positive stimuli, reflecting a negativity bias. Then, negative stimuli associated with visual experience demand more cognitive resources for deep analysis at the post-lexical processing stage. Finally, reaction times for negative words are slower than positive words, indicating a “negative delay” in word recognition. By contrast, positive content facilitates the semantic processing, reflecting a positivity bias or a positivity offset. In our study, positive words had significantly higher valence ratings than negative words, which might have a potential impact on reaction times.

### 4.2. Modality Specificity of Interoception in Emotional Word Processing

Behavioral results demonstrated that interoceptive words had processing advantages over visual words. A marginally significant modality-specific effect was found in the negative context. This indicates processing differences between interoceptive and visual modalities and the effects of sensory experiences on valence processing. As illustrated by Jiang et al. [7], interoceptive and exteroceptive experiences supply the grounding meaning of emotional words, but highlight different conceptualizations of emotions. This linguistic dichotomy may reflect deeper, biologically grounded differences in how emotional experiences are represented and enacted. Correspondingly, we found that interoception plays a distinct role from vision in emotional word processing, which is also in accordance with Connell et al. [8]. In their study, interoception plays a dominant role in representing negative concepts, probably due to the fact that negative words contain richer interoceptive information directly linked to the physiological signals. Our findings extend prior behavioral studies [9,10,11] by clarifying how interoception interacts with valence during emotional words.

ERP results further support the modality-specific effects on valence processing. At the post-lexical stage, a trend toward an interaction between sensory information and valence emerged in the N400 component, with smaller N400 amplitudes for positive interoceptive words relative to positive visual words. The N400 indexes efforts for lexical semantic access and semantic integration [30,47]. Our findings indicate that individuals exerted less effort to retrieve and integrate the semantics of positive interoceptive words than positive visual words. It is probable that positive mood promotes approach motivation and induces flexible cognition [52]. Additionally, prior work emphasized a close relationship between interoception and emotional experience [55,56]. Zaki et al. [56] found that the brain areas relevant to emotional experience overlap with those of interoceptive signals, such as the insular regions. Therefore, combined with our findings, interoceptive information may to some extent promote the semantic processing in a positive context. Furthermore, interoceptive words elicited reduced LPC amplitudes compared to visual words in the negative condition. The LPC reflects the sustained and elaborate processing of emotional information at the late stage, including imagery, semantic elaboration, or spreading activation [14]. The enhanced LPC for negative visual words suggests higher cognitive load during semantic processing, since vision-dominant concepts were strongly correlated with imageability and they require more time to construct and simulate environment details for better understanding. By contrast, words rated with high interoceptive scores were less imageable [57] but contained rich affective information grounded in bodily states, allowing efficient semantic integration of emotional words through embodied simulation. From the perspective of Conceptual Metaphor Theory [58], words and idiomatic phrases are by means of conceptual metaphor or metonymy of embodied experiences or visceral sensations to describe the abstract emotions or affective states for a better understanding of semantic meanings.

Altogether, our study finds shelter in research on modality-specific effects on conceptual representation and highlights the contribution of interoception in emotional word processing. Our findings align with the perceptual symbol system theory [59], which argues that conceptual symbols are grounded in modality-specific perceptual experiences. As linguistic representations develop alongside the perceptual symbols they correspond to, individuals tend to simulate relevant perceptual experiences when processing verbal information. Our empirical findings on sensory modality effects illustrate the embodied nature of human cognition. Both interoceptive and exteroceptive systems contribute to the neural and mental representation of subjective experiences, thereby reinforcing the theoretical framework of interoceptive embodied cognition [60]. Zhou et al. [60] proposed a model where interoception collaborates with the brain to represent emotional concepts. Interoceptive signals offer physiological clues, while cognitive processes (i.e., attention allocation and memory retrieval) help people integrate these signals with environmental information and past experience to construct emotional concepts. In this view, interoception drives emotional conceptualization, and cognitive factors and environmental cues regulate this process.

Significantly, the present study provides empirical support for recent theoretical work (e.g., [23,41,60,61,62]) and addresses unsolved questions of when emotional valence interacts with sensory experiences during processing emotional words and to what extent these words are grounded in interoceptive and visual experiences. It accords well with our expectations and advances understanding of modality-specific features in processing of emotional words. Moreover, this study reveals a dynamic pattern of emotional valence, with a negativity bias at the early attention capture stage and a positivity bias at the behavioral decision stage. To our knowledge, the present study is the first to investigate how sensory information modulates the processing of emotional words within an interoceptive embodied cognition framework, and to examine the relationship between interoception and emotional valence using ERP.

However, several limitations in the current study should be noted. Firstly, marginally significant interactions between sensory information and valence observed in behavioral and neural results may be attributed to insufficient samples of participants and materials. Secondly, considering the research background and experimental design, only interoception and vision modalities were selected to examine the modality-specific effects. Future research would expand the scope of sensory modalities and language, involving auditory, tactile or other exteroceptive modalities, to further investigate modality-specific embodiment in emotional word processing beyond the Chinese language. Finally, this study employed a lexical decision task, which is an implicit measurement with shallow semantic processing. For future studies, some explicit tasks would be conducted to explore the deep semantic processing, such as an emotional categorization task or emotional valence judgment. In addition to the experimental task, the lexical category of stimuli would be further optimized. The emotional adjectives are selected for this study; nouns and verbs would be considered in future work.

## 5. Conclusions

The present ERP study confirmed the modality-specific effects on emotional word processing and elucidated the role of interoception in modulating emotional word processing. To address the debate of emotional polarity, our findings demonstrate a dynamic pattern of valence effects from neural encoding to behavioral responses. Moreover, modality-specific effects on emotional word processing emerge at the post-lexical stage, where sensory experiences modulate the semantic retrieval and integration of emotional words. Both interoceptive and visual information have impacts on valence processing in a modality-specific manner. Remarkably, our study provides empirical support for the modality-specific hypothesis within the framework of interoceptive embodied cognition and offers novel implications for future research on emotional word processing.

## Figures and Tables

**Table 1:** Mean (SD) of various variables across conditions.

Variables	Positive		Negative	
	Interoception	Vision	Interoception	Vision
Sample	兴奋(excited)	辉煌(glorious)	愤怒(angry)	丑陋(ugly)
Strokes	18.07(4.92)	17.17(5.19)	18.13(3.73)	18.87(5.44)
Frequency	2.92(0.77)	2.68(0.63)	2.88(0.55)	2.70(0.61)
Familiarity (1–7)	6.87(0.32)	6.75(0.33)	6.72(0.42)	6.68(0.47)
Concreteness (1–5)	3.38(0.44)	3.40(0.45)	3.39(0.30)	3.21(0.34)
Valence (1–7)	5.64(0.54)	5.84(0.44)	2.50(0.43)	2.46(0.54)
Arousal (0–4)	2.76(0.38)	2.59(0.40)	2.62(0.50)	2.54(0.46)

## Data Availability

Data are available upon reasonable request from the corresponding author due to participants’ privacy.
